# The Alexander Technique and musicians: a systematic review of controlled trials

**DOI:** 10.1186/1472-6882-14-414

**Published:** 2014-10-24

**Authors:** Sabine D Klein, Claudine Bayard, Ursula Wolf

**Affiliations:** Institute of Complementary Medicine, University of Bern, CH-3010 Bern, Switzerland

**Keywords:** Alexander technique, Systematic review, Musician, Performance anxiety, Posture

## Abstract

**Background:**

Musculoskeletal disorders, stress and performance anxiety are common in musicians. Therefore, some use the Alexander Technique (AT), a psycho-physical method that helps to release unnecessary muscle tension and re-educates non-beneficial movement patterns through intentional inhibition of unwanted habitual behaviours. According to a recent review AT sessions may be effective for chronic back pain. This review aimed to evaluate the evidence for the effectiveness of AT sessions on musicians’ performance, anxiety, respiratory function and posture.

**Methods:**

The following electronic databases were searched up to February 2014 for relevant publications: PUBMED, Google Scholar, CINAHL, EMBASE, AMED, PsycINFO and RILM. The search criteria were “Alexander Technique” AND “music*”. References were searched, and experts and societies of AT or musicians’ medicine contacted for further publications.

**Results:**

237 citations were assessed. 12 studies were included for further analysis, 5 of which were randomised controlled trials (RCTs), 5 controlled but not randomised (CTs), and 2 mixed methods studies. Main outcome measures in RCTs and CTs were music performance, respiratory function, performance anxiety, body use and posture. Music performance was judged by external experts and found to be improved by AT in 1 of 3 RCTs; in 1 RCT comparing neurofeedback (NF) to AT, only NF caused improvements. Respiratory function was investigated in 2 RCTs, but not improved by AT training. Performance anxiety was mostly assessed by questionnaires and decreased by AT in 2 of 2 RCTs and in 2 of 2 CTs.

**Conclusions:**

A variety of outcome measures has been used to investigate the effectiveness of AT sessions in musicians. Evidence from RCTs and CTs suggests that AT sessions may improve performance anxiety in musicians. Effects on music performance, respiratory function and posture yet remain inconclusive. Future trials with well-established study designs are warranted to further and more reliably explore the potential of AT in the interest of musicians.

## Background

Music playing-related injuries, stress and performance anxiety are common in music students, professionals as well as amateur musicians. Most frequently reported are musculoskeletal disorders such as back and neck pain, inflammation of the tendon sheets, muscular overuse syndromes and neuropathy in the upper limbs, depending on the instrument played [1, 2]. Systematic reviews found 39-87% [3] and 29-93% [4] of playing-related musculoskeletal disorders in adult orchestral musicians and pianists, respectively. Although the wide prevalence range suggests a heterogeneous definition of disorders included in these studies, it becomes apparent that musculoskeletal disorders represent a major problem among instrumental musicians. Other known disorders in musicians include performance anxiety [5], focal dystonia [6], tinnitus [7], problems of the lips and teeth [8] as well as contact allergies [9].

Musculoskeletal disorders are commonly addressed with physical therapy, but specialised care for musicians is rarely available [10]. Hence, other methods such as Alexander Technique (AT), yoga or Feldenkrais method are also used. AT is a psychophysical method, developed by Frederick Matthias Alexander (1869–1955). It uses enhanced kinaesthetic awareness and voluntary inhibition to prevent non-beneficial movement patterns. The primary focus is put on the relationship between head, neck and back as crucial in effecting an overall integrated pattern of coordinated behaviour. Through this conscious re-education of thinking and moving unnecessary muscle tension is released, which leads to more ease in movement and breathing and a better coordinated “use” (technical AT term describing the manner in which a person moves and behaves). AT is usually taught one-to-one by licensed teachers and combines verbal instructions with hands-on guidance. The psychophysical connection through hands-on work is specific for AT and distinguishes it from bodywork techniques.

According to a recent review, there is good evidence that AT lessons are effective for chronic back pain [11]. Musicians report that AT enables them to move and breathe more easily and thereby improves their quality of music playing [12, 13], but effects of psychophysical therapies are generally not easy to assess objectively. Since AT may in principle constitute a promising method for musicians, an overview of published studies including outcome parameters used and limitations encountered would help to facilitate the design of future studies.

Therefore, the aim of this review was to evaluate systematically the current evidence for the effectiveness of AT sessions (one-to-one or group lessons) for musicians’ health and improved music performance. Since little research on this topic was found searching scientific databases, a broader search strategy had to be used. Therefore, additionally to peer-reviewed articles, master’s and doctoral theses as well as conference proceedings were included.

## Methods

The preferred reporting items for systematic reviews and meta-analyses (PRISMA) guidelines were followed in this review [14]. The review was not registered in any database.

### Literature search

The following electronic databases were searched for relevant publications: PUBMED, CINAHL, EMBASE, AMED, PsycINFO, RILM and Google Scholar. The publication time was from the start of each database up to February 2014. The search criteria were “Alexander Technique” AND music* [all fields], resulting in 97 combinations in PUBMED. Additionally, reference lists were searched, and experts and societies of AT or musicians’ medicine contacted to retrieve further publications.

### Inclusion and exclusion criteria

Prospective studies meeting the following criteria were included: Study participants were musicians (children, adults, amateurs, students or professionals, singers and instrumental musicians). Interventions were AT sessions (one-to-one or group lessons) or exercises based on AT principles. The study had a control group, which either received any other or no control intervention. Any outcomes related to music playing, musicians’ health or posture were accepted. Randomised controlled trials (RCTs) and controlled trials (CTs) were eligible.

All forms of publications were included, e.g. peer-reviewed articles, master’s and doctoral theses, conferences presentations etc.

Studies were excluded if they were not related to musicians, AT was mentioned but not investigated, if the article did not report on the results of an interventional study, if it was a study without control group, or a case report or series.

### Data collection

Data were extracted by one author (CB) and verified by a second author (SDK).

For each study, the data extracted were: study design, publication type, study population, experimental intervention(s) and control intervention(s), outcome measures, main results.

### Assessment of risk of bias

The risk of bias was assessed according to Jadad et al. [15], modified as described in [16] with a maximum possible score of 4 for RCTs.

## Results

**Figure 1:**
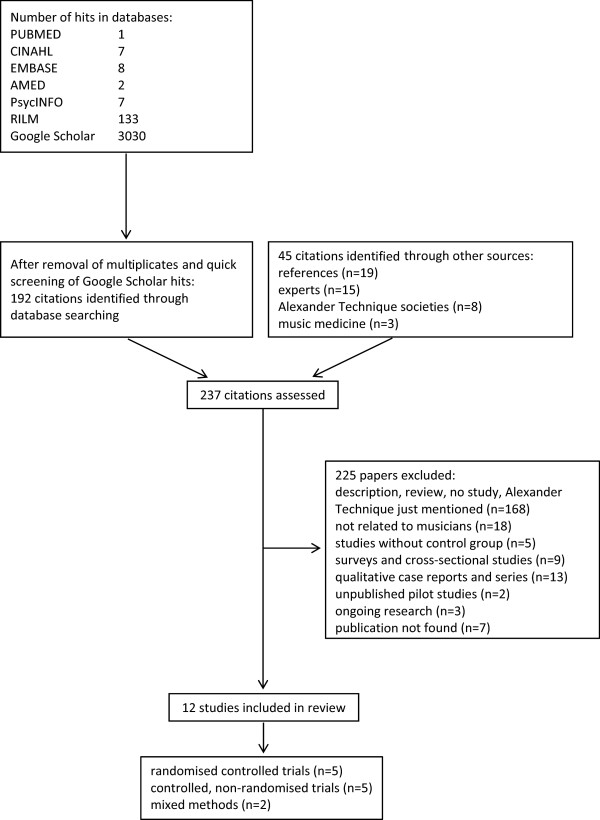
**Study selection.**

Of the 237 citations screened, 225 were excluded (Figure 1). Seven citations were classified as “not found”, because not even an abstract was available [17–23]. Of the 12 studies included in this review, 3 were peer-reviewed publications, 4 doctoral and 3 master’s theses, and 2 appeared in conference proceedings. 5 RCTs, 5 CTs and 2 studies with mixed methods on the effectiveness of AT sessions on music performance, use (and misuse), pain and well-being, posture and performance anxiety are summarised in Table 1. Adverse events of AT sessions were generally not described in the studies.

**Table 1:** **Selected studies (RCTs and CTs) of effectiveness of AT** ^**a**^

Study, type of publication	Design	Participants	Experimental intervention(s)	Control intervention(s)	Outcome measures	Main results
Doyle 1984 [24], PhD thesis	RCT	72	Short hands-on contact with thought: free neck muscles before playing	Short hands-on contact with thought: tighten neck muscles before playing	Head-neck relationship	98.6% of subjects showed changes in the head-neck relationship when taking up their instrument to play.
		Violin players			(Defined as the angle between a line going through the sternal notch and the first dorsal vertebra and a vertical line going through the middle of the chair and measured on photographs)	Release in tension in the neck muscles was closely associated with postural changes towards the grid vertical in 71.4% of subjects (p <0.001). When tightening the neck muscles 94.6% moved in a forward direction (p <0.001).
		42 female, 30 male, age 11–19 years (music schools, school orchestras)				
Dennis 1987 [25], Ed.D. dissertation	RCT	13	AT	None	Music performance (posture, movement, breath control, overall performance) judged by 6 expert observers from video-tapes on a 7-point scale	Control group performed better in maximal voluntary ventilation (t-test, p =0.052); no other significant differences between groups occurred.
		Young adult wind instrument players	20 sessions, 30 min, over 4 months, one-to-one		Respiratory function (standard spirometry, maximal inspiratory and expiratory pressures)	
		8 female, 5 male, age 22–33 years				
Valentine et al. 1995 [26], peer-reviewed	mixed methods:	25	AT	None	Height, peak flow, heart rate	AT group showed improvement relative to control group in musical and technical quality, heart rate variance, self-rated anxiety, and positive attitude to performance (ANOVA, p <0.05).
	- RCT	Music students	15 sessions, one-to-one		Music performance and degree of misuse^b^ rated by 4 blinded expert judges from video-tapes	Effects were mostly restricted to performance in low stress class situations (with the exception of heart rate variance).
	-interviews	21 female, 4 male, age 19–32 years (music department of a university)			Music Performance Anxiety Self-Statement	
					Nowlis mood adjective checklists	
Lorenz 2002 [27], master’s thesis	RCT	22	sensory awareness and body alignment exercises based on AT	None	performance anxiety (degree, symptoms) and effects of AT on performance anxiety measured by 4 questionnaires (designed by author)	Inconclusive effect of exercises on performance anxiety.
		Female choral singers, age 13–16 years (high school)	1 to 4 min exercises, 3 to 4 times weekly, over 13 weeks, group training			
Egner and Gruzelier 2003 [28], peer-reviewed	RCT	61	1. alpha/theta NF	4. Physical exercise	Assessment by 3 expert judges from video-tape in random order on 10-point scales adapted from a standard set of music performance evaluation criteria (overall quality, perceived instrumental competence, musicality/musical understanding and communication)	Significant improvements in music performance occurred in the alpha/theta NF group (p <0.01 for 3 out of 4 criteria; mean improvement rate 12%), but no post-training performance changes in any other group.
		music students	2. beta1 NF	5. Mental skills training	Spielberger’s state-anxiety inventory	Reduction in pre-performance anxiety was observed in all 6 groups (p <0.05).
		43 female, 18 male, mean age 23.1 ± 2.21 years (college)	3. sensorimotor rhythm NF	6. AT: 15 sessions, 30 min, weekly, over 15 weeks, one-to-one		
			10 sessions, 15 min, over 6–8 weeks			
Valentine and Williamon 2003 [29], conference proceedings	RCT	18	AT	alpha/theta NF	Assessment of AT use^b^ by blinded expert on 7-point scale	AT group showed improvement relative to NF group in 7 out of 10 measures of AT use (p <0.05, one-tailed values).
		Music students (college)	12 sessions, 30 min, weekly, one-to-one	10 sessions, 15 min, over 6–8 weeks		
Mozeiko 2011 [30], dissertation	mixed methods:	51	AT	none	Pain, executive skill function, well-being, awareness	Significant changes were found in awareness and executive skill function in AT group compared to control group (MANOVA, p <0.01).
	- RCT	Female violinists and violists, age 18–34 years	20 sessions, 30 min, twice a week, over 10 weeks, one-to-one		- Questionnaires (quantitative, questions from author and previous studies, von Korff scale for pain, 10-point Likert scales)	Convergence of quantitative and qualitative data showed also improvement in pain.
	-Interviews		Lie down in semi-supine position 10–15 min once or twice a day		- Interviews (qualitative)	
Barlow 1956 [31], peer-reviewed	CT	74	AT (“conditioning”)	Verbal instructions, manual adjustment, exercises	Postural faults (according to author’s scoring system)	In the AT group the number of faults decreased from 9 to 4 in women and from 11 to 5 in men. In the control group the number of faults increased from 7.5 to 7.9 in women and from 10.6 to 11.7 in men.^c^
		44 speech and 30 music students				
		42 female, 32 male (college)				
Armstrong 1975 [32], master’s thesis	CT	8	AT	none	Performance anxiety (author’s questionnaire)	AT group experienced less nervousness and stress after training, while there wasno change in the control group.
		Piano students (music department of a university)	4-6 sessions, 30 to 45 min, over 6 weeks, one-to-one		Qualitative observations regarding movement	Video-taping revealed less stiffness and increased flexibility in shoulders and neck in the AT group.^c^
Nielsen 1988 [33], conference proceedings	CT	39	1. AT	2. exercises	heart rate, BP	Exercise group showed
		Professional musicians in orchestra	20 sessions, over 8 weeks, one-to-one	7 km running 3 times a week, over 8 weeks	feedbacks	- significant reduction in heart rate (paired t-test, p <0.05)
				3. beta blocker		- increase in general well-being (responses on qualitative questionnaires).
				40 mg Propranolol, 1.5 h before concert		AT group and beta blocker groups showed
				4. placebo tablet		- significant reduction in systolic BP (p <0.02)
						- significant reduction in increases in systolic BP from final rehearsal to concert (p <0.05).
						Beta blocker group experienced unwanted side effects.
Engelhart 1989 [34], PhD thesis	CT	23	1. AT	2. Progressive muscle relaxation according to Jacobson	Tone quality rated by 3 experts on a 9-point Likert scale	No significant difference was found after interventions among the 3 groups with respect to change in tone quality.
		Beginning singers	10 sessions, 50 min, over 2 weeks, group training	3. Standard vocal exercises	Preparatory muscle activity determined by surface EMG	Group-time interaction effects occurred for 6 of 18 EMG variables (ANOVA, p <0.05); no overall pattern indicated differences between the 3 groups.
		18 females, 5 males, age 18–29 years (students with no previous vocal training)		10 sessions, 50 min, over 2 weeks, group training		
Hoberg 2008 [35], master’s thesis	CT	12	Selected AT principles included in flute lessons (with author)	flute lessons without AT principles (with other teachers)	Performance anxiety (author’s questionnaire):	AT group had decreased performance anxiety.^c^
		flute students with performance anxiety	18 months	18 months	- degree	
		age 11–18 years			- symptoms	

^a^ *AT* Alexander Technique, *RCT* randomised controlled trial, *CT* controlled trial, *BP* blood pressure, *NF* neurofeedback, *EMG* electromyography.

^b^“Use” is an AT term which characterises the manner in which a person moves and behaves while doing something. It is influenced by thinking and emotions and affects the functioning of the whole person.

^c^No significance testing was performed for these results.

### Randomised controlled trials and mixed methods studies

Doyle investigated in his PhD thesis the changes in posture and especially in the head-neck relationship of violinists while playing their instrument [24]. The experimental interventions consisted of short hands-on contact, while one group was asked to think of freeing the sternocleidomastoid muscles, so that the skull moved away from the experimenter’s hand (forwards and up) and the other group was asked to think of tightening the muscles, so that the skull moved towards the experimenter’s hand (back and down). Photographs were taken before the subjects were given their instrument, when holding their instrument and after the short interventions. It was found, that a change in the head-neck relationship occurred when violinists take up their instrument to play, and that a release in tension in the neck muscles was closely associated with postural changes towards the grid vertical. This study did not evaluate the effectiveness of AT sessions, but the immediate effect on posture when subjects were given instructions similar to the initial guidance given by AT teachers. A source of bias in this study was the evaluation of the photographs by the author, who also provided the interventions.

Dennis hypothesised that 20 AT lessons would result in functional improvements in respiratory function and musical performance in young adult wind instrumentalists [25]. When mean difference scores (post – pre) between the AT and the control group were compared, the control group showed better performance in maximal voluntary ventilation than the AT group. No other significant differences in respiratory functions were found. The subjects in the AT group submitted short reports on their experience of the experimental procedure, which suggested a positive effect of the AT lessons. However, no significant differences between AT and control group were shown in the variables rated by the experts judging music performance. The author assumed some degree of subject bias, since blinding of the participants was not possible.

Valentine et al. conducted a mixed methods study with quantitative and qualitative measures to investigate the effects of AT sessions on performance anxiety and music performance [26]. Before and after the course of AT sessions, a variety of measures (e.g. heart rate, music performance, misuse, performance anxiety) were taken in both high (audition, recital) and low stress situations (performance in class). The AT group showed improvements relative to the control group in overall musical and technical quality, heart rate variance, self-rated anxiety and positive attitude to performance. Peak flow as a measure of respiratory function did not significantly improve in the AT group relative to the control. Interviews of the AT group revealed that participants had increased awareness of tension and improved ability to relax. Since these effects were mostly restricted to performance in the low stress situation, it was concluded, that 15 AT lessons were insufficient to develop a level of skill required to apply the technique in high stress situations, or that an enhanced ability to deal with performance anxiety was not the main benefit from AT.

Performance anxiety in 2 high school choirs was investigated by Lorenz [27]. Short sensory awareness and body alignment exercises based on AT were included over 13 weeks in the vocal warm-up of one choir. Degree and symptoms of performance anxiety were assessed in 22 female students by questionnaires pre- and post-exercises, prior to 2 similar school performances. Symptoms of performance anxiety were frequently experienced, and the effect of AT based exercises was inconclusive. In this study, only short exercises based on AT but not AT sessions were provided by the author, who was not a certified AT teacher. The questionnaires to assess performance anxiety were designed by the author and were not validated instruments.

Egner and Gruzelier investigated the effects of 3 neurofeedback (NF) protocols and 3 control interventions (physical exercise, mental skills training, AT) on music performance and performance anxiety [28]. 61 music students were randomly allocated to 1 of 6 training groups. The students performed two musical pieces of their own choice before and after the interventions. The alpha/theta NF group showed significant improvements in music performance, while no significant changes were observed in any of the other groups. A reduction in pre-performance anxiety was observed in all 6 groups after training. The main focus in this study lay on NF, while AT was regarded as control intervention. 15 AT sessions may have been again insufficient to evoke an effect on criteria such as e.g. overall quality of music performance or musical understanding. NF and AT training was further compared by Valentine and Williamon regarding AT use [29]. In 7 out of 10 measures of AT use, there were significant interactions between group (AT, NF) and training (before, after). The AT group showed improvements, while the NF group declined. According to the students’ feedback AT training was perceived as highly beneficial and satisfactory.

In a mixed methods study using questionnaires, observations and semi-structured interviews, Mozeiko investigated the effects of AT sessions on 4 variables in female violinists and described the experiences of the participants [30]. Outcome measures were awareness, executive skill function, pain and well-being, which were assessed before and after the intervention. The experimental group showed statistically significant improvements in awareness and executive skill function compared to the control group. For pain statistical significance was observed, yet after removing outlying scores to achieve a normal distribution these changes between groups were no longer significant. In contrast to previous studies, participants had more AT sessions and were additionally instructed to lie down in a semi-supine position once or twice a day, which was considered an integral part of learning the AT. All outcome parameters were subjective and assessed by questionnaires.

### Controlled trials

Barlow, a pioneer of AT studies, compared in a study in 1956 postural faults of male and female speech and music students from 2 schools [31]. While the students from the Central School of Speech were given verbal instructions and occasional manual adjustments, the students from the Royal College of Music were trained by AT (described in the article as “a conditioning procedure”). From beginning to end of the training, the number of postural faults defined by the author in the group receiving verbal instructions slightly increased, while in the AT group the number of faults decreased. No statistical analysis was performed, and according to present standards, the study is highly biased.

Armstrong hypothesised that AT sessions would help pianists to cope with stress and nervousness [32]. In the experimental group, less overall stiffness was observed in the second video-taping after AT lessons compared to the one at the beginning. The results of the questionnaires indicated a general decrease in nervousness from pre- to post-treatment performance in all subjects of the AT group whereas the control group considered the nervousness in performance to be as much of a problem as it had been in the first situation. All 4 subjects of the experimental group reported that they wanted to continue with the AT work. The number of participants as well as the number of AT sessions was found to be insufficient to draw reliable conclusions. The study is highly biased, since AT sessions were given by the author, who also assessed the video-tapes.

In another controlled trial about performance anxiety by Nielsen [33], 39 musicians were divided into 4 groups (AT, running, beta blocker, placebo tablet). Outcome measures were heart rate and blood pressure (BP). The running group showed a significant reduction in heart rate and an increase in general well-being. The AT and beta blocker groups showed a significant reduction in systolic BP. The placebo group showed no significant changes. Participants in the beta blocker group experienced unwanted side effects such as feeling indifferent or cold, whereas participants in the AT group reported improvements in breathing and surplus energy and in the exercise group an increased well-being. The advantages of this study were objective measurements for stress and the comparison with the application of a beta blocker.

Engelhart tried to determine the impact of preparatory muscle activity (so-called “preparatory set”, which is preceding voluntary movement) in singing and to correlate changes in the preparatory set behaviour and improved singing quality [34]. Each participant performed a song before and after training (AT, progressive muscle relaxation according to Jacobson, or standard vocal exercises). These performances were video-taped and rated in random order by 3 expert judges, who rated the tone quality on a 9-point scale. Preparatory muscle activity was determined by surface electromyography (EMG) in 4 participants of each group. No significant differences in change of tone quality were found between groups; therefore, there were no correlations to changes in the preparatory set. It was concluded, that the sample size was too small and the length of the training period was too short to detect significant differences.

Hoberg investigated if performance anxiety could be reduced by implementing principles of AT in flute teaching [35]. One group of 6 students received lessons with a teacher who applied selected principles of AT, the other 6 students had lessons with other teachers, who did not apply AT principles. Comparison of both groups at the end of the study revealed that both groups experienced nervousness, but the participants in the AT group were less nervous than at the previous examination. Comparing the symptoms, the control group had more anxiety symptoms than the AT group. In this study, the participants did not receive AT sessions, but selected AT principles were applied by one of the music teachers who was also the author of this study and not a registered AT teacher. The questionnaires to assess performance anxiety were designed by the author and were not validated instruments. No statistical tests were performed to compare the two groups or pre- and post-results.

### Summary of risk of bias

The risk of bias of the individual studies is summarised in Table 2. Blinding of the participants was impossible due to the nature of the intervention (except in [24]), therefore, a modified Jadad score was used [15, 16]. Randomisation procedures were only described in 2 of the RCTs. 6 out of 12 studies had one or several active control groups (e.g. NF, physical exercise, verbal instructions).

**Table 2:** **Risk of bias in the selected studies**

Study	Described as randomised	Randomisation method described and appropriate	Assessor unaware of group allocation of subjects	Description of withdrawals and drop-outs	Score [15, 16]	AT teacher
Doyle 1984 [24]	Yes	Yes	No	Yes	3	Author
Dennis 1987 [25]	Yes	No	Yes	Yes	3	7 certified AT teachers (not including author)
Valentine et al. 1995 [26]	Yes	No	Yes	Yes	3	2 teachers employed for the study
Lorenz 2002 [27]	Yes	No	Yes (questionnaires)	Yes	3	Author (without professional certification in AT)
Egner and Gruzelier 2003 [28]	Yes	No	Yes	No	2	Not described
Valentine and Williamon 2003 [29]	Yes	No	Yes	No	2	Qualified AT teacher
Mozeiko 2011 [30]	Yes	Yes	Yes (questionnaires)	Yes	4	9 certified AT teachers
Barlow 1956 [31]	No	-	No	No	0	Author (AT teacher)
Armstrong 1975 [32]	No	-	No	Yes	1	Author (qualified AT teacher)
Nielsen 1988 [33]	No	-	Yes	Yes	2	Professional AT teacher
Engelhart 1989 [34]	No	-	Yes	Yes	2	Certified AT teacher
Hoberg 2008 [35]	No	-	Yes (questionnaires)	No	1	Author (not a registered AT teacher)

Investigators were involved in AT teaching in 5 of the studies, while 5 studies used external teachers and 2 gave no information on this point. Main outcomes were assessed by authors in 3 studies (investigating posture or movement) and by external experts, who were blinded to the assignment to AT or control group of the participants in 5 studies. 3 studies used only questionnaires and 1 only physiological measures.

## Discussion

Posture and movements of a musician influence the sound of his/her instrument or voice. One goal of AT lessons is to create ease and freedom with movement, another that musicians overcome habitual postures that predispose them to injuries or decreased function [36]. The underlying idea of several studies presented here was, that AT lessons would result in an improvement of posture and in turn in music playing.

### Summary of evidence

The most commonly investigated outcomes of AT sessions in clinical trials with musicians were music performance, use, performance anxiety and respiratory function. Music performance was investigated in 3 RCTs and 1 CT and assessed by external experts. Only in 1 RCT the musical quality improved in the AT group while it declined in the control group from pre- to post-training [26]. In a study comparing NF to AT, alpha/theta NF sessions but not AT sessions improved the music performance [28]. Possible reasons for the inconclusive results may be (i) music performance being a complex process and as such not easy to assess, and (ii) a study duration of 3 to 4 months being too short for the participants to incorporate the skills acquired during AT lessons into their music playing in a way that music performance was observably improved.

Posture, head-neck relationship, movement, effects of AT sessions on use or misuse were investigated in 4 RCTs and 2 CTs. In most RCTs, effects of AT sessions on use ore misuse were inconclusive, yet 1 RCT with defined rating scales reported improved use in the AT group relative to the NF group [29]. One inconclusive RCT reported a very low inter-rater agreement for misuse by 2 AT experts [26], indicating that it may be difficult to assess use or misuse from video-tapes.

Performance anxiety was an outcome in 3 RCTs and 3 CTs and improved by AT in all but 1 RCT, in which not one-to-one sessions but only short group exercises bases on AT were provided [27]. While most studies used the Spielberger’s state-anxiety inventory or self-designed questionnaires, in 1 CT heart rate and BP were measured [33]. In studies where AT was compared to active control interventions (e.g. NF or exercise), these other interventions were also effective in reducing performance anxiety. It has been shown, that other interventions such as yoga or physical activity can positively influence performance anxiety [37, 38].

No improvements were found in respiratory function in 2 RCTs. In 1 of these wind instrumentalists were investigated [25], who had probably already been training and optimising their breathing [39]. The other study found a non-significant directional effect of improvement in peak flow in the AT compared to the control group [26]. In contrast, a study in healthy volunteers found significant increases in peak expiratory flow, maximal voluntary ventilation, and maximal inspiratory and expiratory mouth pressure after 20 AT lessons [40]. However, compared to the control group, these changes were not significant.

The effects of AT on music playing may be multifaceted, as shown in the various outcome parameters chosen. When subjective measures such as music performance were applied, usually several external experts were involved [28], and sometimes they were trained before [25] or their agreement was determined [26, 34].

Participants in the studies included in this review were mostly healthy and young, in contrast to other studies with people suffering from Parkinson’s disease [41] or back pain [42], for which AT lessons were effective. Positive results for the effectiveness of AT lessons were obtained for performance anxiety, the only medical condition investigated. None of the studies focused on musicians with pain conditions.

### Limitations

The number of participants was rather low in most studies and ranged from 13 to 72 in RCTs (average 37), from 8 to 74 in CTs (average 31). Statistical tests were not performed in some of the studies. Conference abstracts were limited in word count and did not adequately describe all methods and results.

AT is usually taught in one-to-one sessions, and since it is considered a re-educational method, effects would only be expected after several sessions. In the trials included in this review, one-to-one lessons as well as group training were applied, and training varied from a few minutes to 20 sessions of 30 minutes. A few minutes of group training over several weeks seemed to be insufficient to reduce performance anxiety in female choral singers [27], while 15 one-to-one sessions improved performance anxiety in 2 RCTs [26, 28]. Although it could be assumed that outcome measure evaluations were performed shortly after the end of the last AT lesson, most studies were not explicit with regard to the timing.

In this review, amateur musicians, music students and professionals were included. The most often assessed parameter, performance anxiety, seemed to improve by AT sessions in all groups of musicians, but the majority of studies were carried out with music students. The 2 studies with amateurs (children) assessed AT-based exercises rather than AT sessions, that were provided by teachers without professional AT certification [27, 35]. Respiratory function was investigated in 2 studies, one with young adult wind instrument players and one with music students from a university, and AT lessons did not improve the outcome in either study. Overall, no comparisons between the groups of musicians and their response to AT lessons can be drawn.

Adverse events (or their absence) were also not described, but AT is considered a low risk method due to the movements that are in range of normal movements. In a back pain trial comparing AT, exercise and massage, none of the 288 patients in the AT groups reported adverse events [42]. Generally, AT is regarded a safe method for which no serious side effects have been reported [43].

This review reported mostly on quantitative data, although 2 studies used a mixed design including interviews. Insufficient reporting of data (without standard deviations or standard errors of the mean) made it impossible to report effect sizes or to conduct a meta-analysis for music performance or performance anxiety.

Additionally to searching databases for studies on AT we contacted experts and societies of AT and musicians’ medicine. Nevertheless, we cannot exclude that studies on the effectiveness of AT on musicians’ performance or health may have been missed. Seven studies including the respective abstracts were not available [17–23], and thus it remains unclear, whether they had met the inclusion criteria.

The reported outcomes were manifold, ranging from physiological measures (e.g. BP) over self-reported performance anxiety to music performance judged by external experts. The study participants ranged from schoolchildren to professional musicians in an orchestra. Thus, drawing overall conclusions about the effectiveness of AT lessons for musicians was difficult. Nevertheless, this review has relevant implications and its strength lies in presenting the current state of research in this area.

## Conclusions

A variety of study designs and outcome measures has been used to investigate the effectiveness of AT sessions in musicians. Evidence from RCTs and CTs suggests that AT sessions may improve performance anxiety in musicians. Effects on music performance, use and respiratory function yet remain inconclusive. Future trials with well-established study designs, a sufficient number of participants and subjective as well as objective outcomes are warranted to further and more reliably explore the potential of AT as a low risk method in the interest of musicians.

## Abbreviations

AT: Alexander Technique
BP: Blood pressure
CT: Controlled trial
EMG: Electromyography
NF: Neurofeedback
RCT: Randomised controlled trial.

## References

[1] Leaver R, Harris EC, Palmer KT (2011). Musculoskeletal pain in elite professional musicians from British symphony orchestras. Occup Med (Lond).

[2] Ackermann B, Driscoll T, Kenny DT (2012). Musculoskeletal pain and injury in professional orchestral musicians in Australia. Med Probl Perform Art.

[3] Zaza C (1998). Playing-related musculoskeletal disorders in musicians: a systematic review of incidence and prevalence. CMAJ.

[4] Bragge P, Bialocerkowski A, McMeeken J (2006). A systematic review of prevalence and risk factors associated with playing-related musculoskeletal disorders in pianists. Occup Med (Lond).

[5] Wesner RB, Noyes R, Davis TL (1990). The occurrence of performance anxiety among musicians. J Affect Disord.

[6] Rietveld AB, Leijnse JN (2013). Focal hand dystonia in musicians: a synopsis. Clin Rheumatol.

[7] Toppila E, Koskinen H, Pyykkö I (2011). Hearing loss among classical-orchestra musicians. Noise Health.

[8] Rodríguez-Lozano FJ, Sáez-Yuguero MR, Bejmejo-Fenoll A (2011). Orofacial problems in musicians: a review of the literature. Med Probl Perform Art.

[9] Gasenzer ER, Neugebauer EA (2012). Contact allergies in musicians. Dtsch Med Wochenschr.

[10] Guptill CA (2011). The lived experience of professional musicians with playing-related injuries: a phenomenological inquiry. Med Probl Perform Art.

[11] Woodman JP, Moore NR (2012). Evidence for the effectiveness of Alexander technique lessons in medical and health-related conditions: a systematic review. Int J Clin Pract.

[12] Bosch AJ (2005). Master’s Dissertation. The use of the Alexander Technique in the Improvement of Flute Tone.

[13] Kaplan I (1994). PhD Thesis. The Experience of Pianists who have Studied the Alexander Technique: six case Studies.

[14] Moher D, Liberati A, Tetzlaff J, Altman DG (2009). Preferred reporting items for systematic reviews and meta-analyses: the PRISMA statement. PLoS Med.

[15] Jadad AR, Moore RA, Carroll D, Jenkinson C, Reynolds DJ, Gavaghan DJ, McQuay HJ (1996). Assessing the quality of reports of randomized clinical trials: is blinding necessary?. Control Clin Trials.

[16] Ernst E, Canter PH (2003). The Alexander Technique: a systematic review of controlled clinical trials. Forsch Komplementärmed Klass Naturheilkd.

[17] Calvert C (2006). Unpublished Undergraduate Thesis. Investigating the use of the Alexander Technique to Improve Musical Performance.

[18] Fletcher N (2005). Unpublished Master’s Thesis. Improvement in Musical Performance due to Application of Alexander Technique.

[19] Roberts N, Williamson A (2006). Measurement Science and the Alexander Technique. 6th International Conference for Alexander Teachers Working in Music Institutions: 20–21 February 2006; Royal Northern College of Music, Manchester.

[20] Head S (1996). Master’s Thesis. How the Alexander Technique Informs the Teaching of Singing: the Personal Experience of and Analysis by a Singing Teacher.

[21] Huttlin EJ (1982). PhD Thesis. A Study of Capacities In Wind Instrumentalists and Vocalists.

[22] Hamilton B (1986). Doctoral Dissertation. The Alexander Technique: A practical application to upper string playing.

[23] Richter E (1974). Dissertation. The application of the Alexander Technique to cello playing.

[24] Doyle G (1984). PhD thesis. The task of the Violinist: Skill, Stress and the Alexander Technique.

[25] Dennis RJ (1987). Ed.D. dissertation. Musical Performance and Respiratory Function in Wind Instrumentalists.

[26] Valentine ER, Fitzgerald DFP, Gorton TL, Hudson JA, Symonds ERC (1995). The effect of lessons in the Alexander technique on music performance in high and low stress situations. Psychol Music.

[27] Lorenz SR (2002). Master’s Thesis. Performance Anxiety within the Secondary Choral Classroom: Effects of the Alexander Technique on Tension in Performance.

[28] Egner T, Gruzelier JH (2003). Ecological validity of neurofeedback: modulation of slow wave EEG enhances musical performance. Neuroreport.

[29] Valentine ER, Williamon A, Kopiez R, Lehmann AC, Wolther I, Wolf C (2003). Alexander Technique and Music Performance: Evidence for Improved “use”. Proceedings of the 5th Triennial ESCOM Conference: 8–13 September 2003; Hanover.

[30] Mozeiko KJ (2011). Dissertation. The Effects of Participation in the Alexander Technique on Female Violinists and Violists: a Mixed Methods Study.

[31] Barlow W (1956). Postural deformity. Proc Roy Soc Med.

[32] Armstrong J (1975). Master’s Thesis. Effects of the Alexander principle in dealing with stress in music performance.

[33] Nielsen M, Stevens C (1988). A study of Stress Amongst Professional Musicians. Proceedings of the conference of The Alexander Technique: Medical and Physiological Aspects: 29 November 1987; Aalborg.

[34] Engelhart RJ (1989). PhD Thesis. An Electromyographic Study of Preparatory set in Singing as Influenced by the Alexander Technique.

[35] Hoberg A (2008). Master’s Thesis. Reducing performance anxiety in woodwind playing through the application of the Alexander Technique principles.

[36] Schlinger M (2006). Feldenkrais method, Alexander technique, and yoga – body awareness therapy in the performing arts. Phys Med Rehabil Clin N Am.

[37] Khalsa SB, Butzer B, Shorter SM, Reinhardt KM, Cope S (2013). Yoga reduces performance anxiety in adolescent musicians. Altern Ther Health Med.

[38] Rocha SF, Marocolo M, Corrêa EN, Morato GS, da Mota GR (2014). Physical activity helps to control music performance anxiety. Med Probl Perform Art.

[39] Zuskin E, Mustajbegovic J, Schachter EN, Kern J, Vitale K, Pucarin-Cvetkovic J, Chiarelli A, Milosevic M, Jelinic JD (2009). Respiratory function in wind instrument players. Med Lav.

[40] Austin JH, Ausubel P (1992). Enhanced respiratory muscular function in normal adults after lessons in proprioceptive musculoskeletal education without exercises. Chest.

[41] Stallibrass C, Sissons P, Chalmers C (2002). Randomized controlled trial of the Alexander technique for idiopathic Parkinson’s disease. Clin Rehabil.

[42] Little P, Lewith G, Webley F, Evans M, Beattie A, Middleton K, Barnett J, Ballard K, Oxford F, Smith P, Yardley L, Hollinghurst S, Sharp D (2008). Randomised controlled trial of Alexander technique lessons, exercise, and massage (ATEAM) for chronic and recurrent back pain. BMJ.

[43] Ulbricht C (2011). Parkinson’s disease: an integrative approach. Alternat Complement Ther.

[CR44] The pre-publication history for this paper can be accessed here: http://www.biomedcentral.com/1472-6882/14/414/prepub

